# Plasma levels of coagulation factors VIII and IX and risk of venous thromboembolism: Systematic review and meta-analysis

**DOI:** 10.1016/j.thromres.2023.06.026

**Published:** 2023-06-28

**Authors:** Gordon Lowe, Olivia Wu, Astrid van Hylckama Vlieg, Aaron Folsom, Frits Rosendaal, Mark Woodward

**Affiliations:** a Institute of Cardiovascular and Medical Sciences, University of Glasgow, Glasgow, UK; b Health Economics and Health Technology Assessment Research Unit, University of Glasgow, Glasgow, UK; c Department of Clinical Epidemiology, Leiden University Medical Center, the Netherlands; d Division of Epidemiology and Community Health, University of Minnesota, Minneapolis, USA; e The George Institute for Global Health, University of New South Wales, Sydney, Australia; f The George Institute for Global Health, Imperial College London, UK

**Keywords:** Factor VIII, Factor IX, Meta-analysis, Systematic review, Venous thromboembolism

## Abstract

**Introduction:**

The associations of plasma factor VIII (FVIII) and factor IX (FIX) levels with risk of venous thromboembolism (VTE) are not well defined. We performed a systematic review and meta-analysis of these associations.

**Methods:**

Random effects inverse-variance weighted meta-analysis was used to estimate pooled odds ratios for comparisons across equal quartiles of the distributions and 90 % thresholds (higher versus lower), and for testing linear trends.

**Results:**

Among 15 studies (5327 cases) the pooled odds ratio of VTE for the fourth versus first quarter was 3.92 (95 % confidence interval 1.61, 5.29) for FVIII level; and among 7 studies (3498 cases) 1.57 (1.32, 1.87) for FIX level. Comparing factor levels above, versus below, the 90th percentile, the estimated pooled odds ratios were 3.00 (2.10, 4.30) for FVIII; 1.77 (1.22, 2.56) for FIX; and 4.56 (2.73, 7.63) for both FVIII and FIX considered jointly.

**Conclusions:**

We confirm increases in risk of VTE across population distributions of FVIII and FIX levels. Levels above the 90th percentile have almost twice the risk for FIX level compared to levels below; three-fold risk for FVIII level; and almost five-fold risk for both FVIII and FIX levels elevated.

## 1. Introduction

Venous thromboembolism (VTE): deep vein thrombosis of the lower limb (DVT) with or without pulmonary embolism, is a common disease, affecting 1–2 in 1000 people per year in developed countries [1,2]. Major chronic risk factors include age, male sex, and obesity. In contrast to arterial cardiovascular diseases, VTE shows weak associations with smoking, blood pressure and blood lipids [1–3]. Its multifactorial pathophysiology includes genetic thrombophilias; and elevated plasma levels of coagulation factors [4] including Factors VIII and IX.

With regard to pathophysiology, population studies have shown associations of plasma levels of FVIII and FIX with the major risk factors for VTE: age, male sex, oral hormone use in women, obesity and metabolic syndrome [5–8]. FVIII and FIX levels are also associated with increased plasma levels of coagulation activation markers, which may promote thrombogenesis [6]. FVIII and FIX are also inflammatory reactant proteins, hence are associated with acute and chronic inflammatory diseases, including autoimmune diseases, as part of the inflammatory response.

Since the first reports, between 1990 and 2000, of associations of VTE risk with higher plasma levels of FVIII [9–13] and FIX [14,15], there have been many subsequent publications. In the recent large MEGA study of all coagulation factor levels and VTE risk, Factor VIII and its carrier protein, von Willebrand factor (VWF), showed the strongest associations [16]. In a mediation analysis, adjustment for these two factors reduced the risks of other coagulation factors to unity, with the exceptions of Factor IX and Factor XI [16].

With regard to genetics, a direct causal effect of the FVIII/VWF complex on risk of VTE is supported by a systematic review and meta-analysis of studies of ABO(H) blood group non–O, which confers higher levels of FVIII and VWF than blood group O [17]. This analysis reported a pooled odds ratio of 1.79 (95%CI 1.56, 2.05) for VTE risk in non-O blood groups compared to group O.

To our knowledge, there has been no published formal systematic review and meta-analysis of the associations of FVIII and FIX levels with risk of VTE. We therefore performed the current review and meta-analysis.

## 2. Materials and methods

### 2.1. Study design

We registered our review and protocol on the PROSPERO website, University of York, UK (www.york.ac.uk/inst/crd; registration number CRD42020204122). We performed searches of PubMed and Cochrane databases for population-based studies in adults (aged 16 years or over) from 1990 in English language, reporting on estimates (and measures of variability) of associations of plasma levels of FVIII or FIX with risks of VTE (as diagnosed using any recognised diagnostic criteria). Search terms are detailed in Supplemental Table 1. The date of last search was 2 May 2022. We searched the reference lists of articles identified (including review articles) for additional relevant studies. In the case of multiple publications, the most up-to-date or comprehensive information was extracted. Selection of articles was performed independently by GL and OW, and discrepancies resolved by consensus.

Data extraction was performed by MW and GL, with any disagreements resolved by OW. Risk of bias was scored by OW and GL using the Ottawa-Newcastle risk of bias tool, at the study level, with any disagreements resolved by MW. No study was excluded from meta-analysis on the basis of this risk of bias assessment. For each primary comparison of FVIII or FIX and VTE risk, we required at least 5 studies with comparable relative risks (or hazard ratios, rate ratios or odds ratios) together with a measure of their variability.

### 2.2. Statistical methods

A priori goals were to obtain pooled estimates of relative risk, comparing (for each of FVIII and FIX): for equal quarters of the distribution (taking the first quarter as the reference); for one standard deviation (SD) higher level, where the SD is study- and factor-specific; for a 10 IU/dl higher level; and for those with values higher than, to lower than, the 90 % percentile of the distribution. In addition, we compared those with values higher than the 90 % percentile of the distribution for both factors to those below for each factor (joint association).

In deriving results from published data, the pre-defined inclusion criterion was to reject any result for FVIII that was not clearly adjusted by (or matched for) age and sex (except where single sex). For FIX, published results had to also be adjusted for body mass index (BMI), because past studies have found this to be a particularly important confounder [7,8,18]; use of anticoagulant drugs at baseline was another exclusion. Where qualifying published studies provided results by quarters; per one SD increase; or values higher than, to lower than, the 90 % percentile; they were included in these analyses. Where they provided results across ordinal groups, but not on a continuous scale, an approximate method [19] was used to estimate the odds ratio per 10 IU/dl.

All studies of FVIII except ARIC and CHS (previously published together as LITE) [18,20] and MESA [21] used the case-control design; and all studies of FIX used a case-control design (nested in ARIC, CHS and MESA). Hazard ratios from Cox models were taken from ARIC, CHS and MESA for FVIII, as the preferred estimate of relative risk, and odds ratios from logistic regression in all other cases. For all the analyses specified above, results were pooled, for both factors, using inverse-variance weighted random effects meta-analysis. We interpreted the results using forest plots and I-squared statistics and Q tests for exploring heterogeneity. For simplicity, the term ‘odds ratio’ is used throughout, except when comparing odds ratios and hazard ratios in a sensitivity analysis for FVIII.

For the seven studies represented by the authors - LETS [10,14], OxGlas [15], MEGA [16], LITE (CHS and ARIC studies) [18,20], MESA [21] and AT-AGE [22] - the respective study teams conducted specific analyses – for the four above aims - adjusting odds ratios for age, sex, BMI, and ethnicity where possible. The estimates of joint, FVIII and FIX, effects around their 90 % percentiles were obtained from logistic regression models fitting both main effects as well as the multiplicative interaction.

We also assessed the degree to which their associations with VTE risk were attributable to inflammation, as measured by other inflammatory markers.

## 3. Results

### 3.1. Selection of published reports

We excluded: less informative reports of same study; prospective studies of VTE recurrence, or of survival; studies of post-thrombotic syndrome or thrombotic pulmonary hypertension; studies of VTE in cancer patients only; and studies of cerebral, retinal, upper limb, or portal venous thrombosis.

We selected 24 studies for meta-analysis: 15 of FVIII (including 5327 VTE cases); and 7 of FIX (including 3498 VTE cases). Details of studies are shown in Table 1, including whether FVIII or FIX was measured as activity (FVIII:C, FIX:C) or antigen (FVIII:Ag, FIX:Ag). Details of the selection process are shown in Supplemental Figs. 1 and 2.

In the following paragraphs, we report that all of our analyses showed increases in risk with increasing levels of FVIII or FIX, whether analysed by quarters; by one standard deviation higher factor level; per 10 IU/dl higher factor level; or by greater than 90th percentile threshold versus less.

### 3.2. Analysis by quarters

**Fig. 1.**
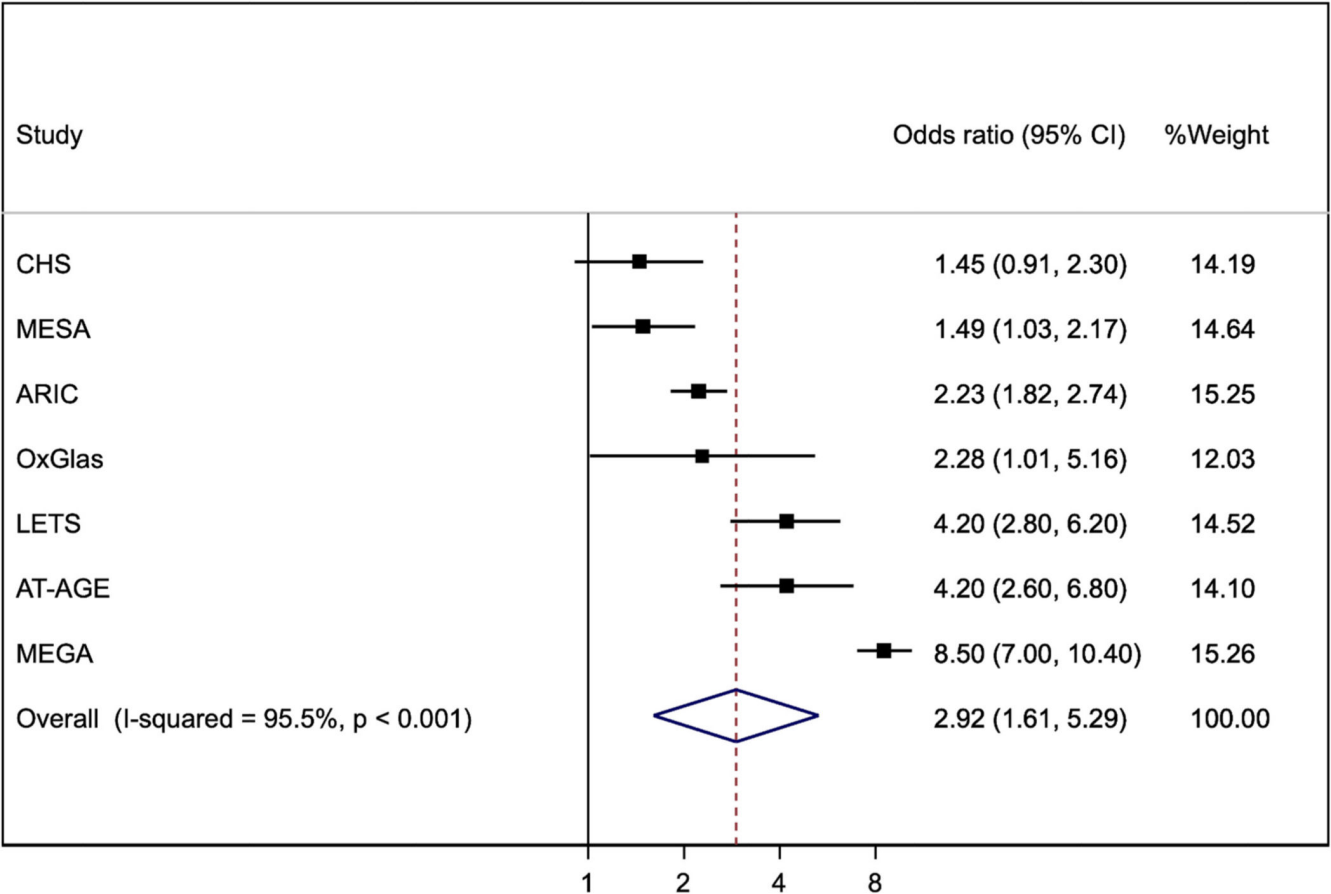
Odds ratios (95%CI) for fourth versus first quarter of FVIII (except that results for CHS, ARIC and MESA are hazard ratios). All results are adjusted for age, sex and BMI, except that OxGlas (women only) adjusted for age and BMI.

**Fig. 2.**
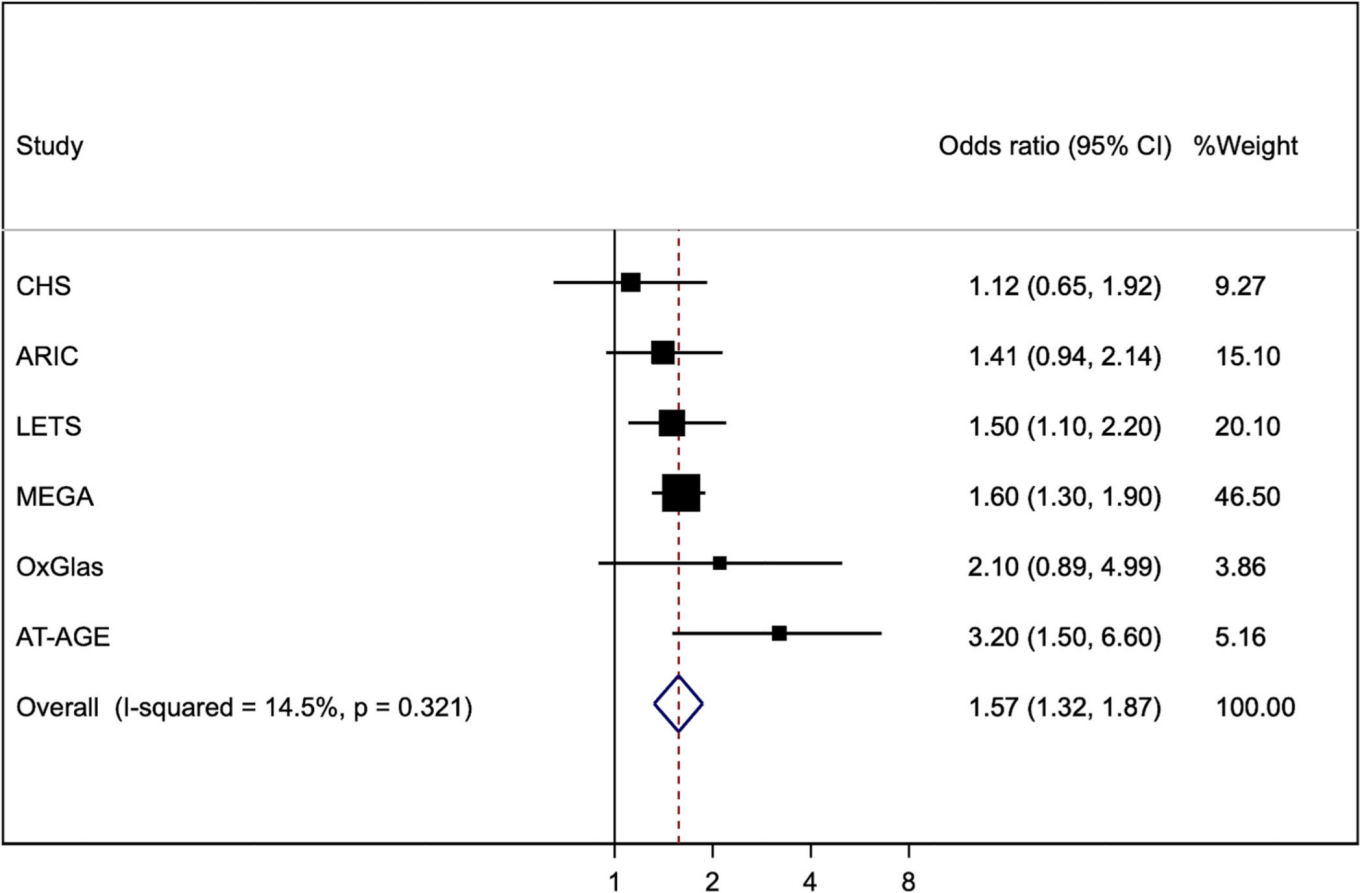
Odds ratios (95%CI) for fourth versus first quarter of FIX. All results are adjusted for age, sex and BMI, except that OxGlas (women only) adjusted for age and BMI.

We performed this analysis in three cohort studies (ARIC and CHS, published together as LITE [18,20]; and MESA [21] and four case-control studies (LETS [10,14], MEGA [16], AT-AGE [22] and OxGlas [15]). The pooled odds ratio for the fourth versus first quarter of FVIII was 2.92 (95 % confidence interval 1.61, 5.29) (Fig. 1). The pooled odds ratio of VTE for the fourth versus first quarter of FIX was 1.57 (1.32, 1.87) (Fig. 2). Heterogeneity between studies was greater for FVIII than for FIX.

Supplemental Table 2 shows details of the 25th, 50th, 75th and 90th percentiles of FVIII and FIX levels in these studies. The pooled odds ratios for VTE by quarter are shown in Supplemental Figs. 3 and 4. There was strong evidence of linearity for FVIII; but weaker evidence for FIX: the odds being fairly constant across the first three quarters, rising in the top quarter.

### 3.3. Analysis per one standard deviation higher factor level

We performed this analysis for FVIII in the seven studies noted above, and in four other published studies [12,23,24]. The pooled odds ratio was 1.46 (1.25, 1.71). Supplemental Fig. 5 shows the odds ratios in individual studies.

We performed this analysis for FIX in six of the seven studies noted above, and in one other published study [24]. The pooled odds ratio was 1.19 (1.11, 1.27). Supplemental Fig. 6 shows the odds ratios in individual studies.

### 3.4. Analysis per 10 IU/dl higher factor level

**Fig. 3.**
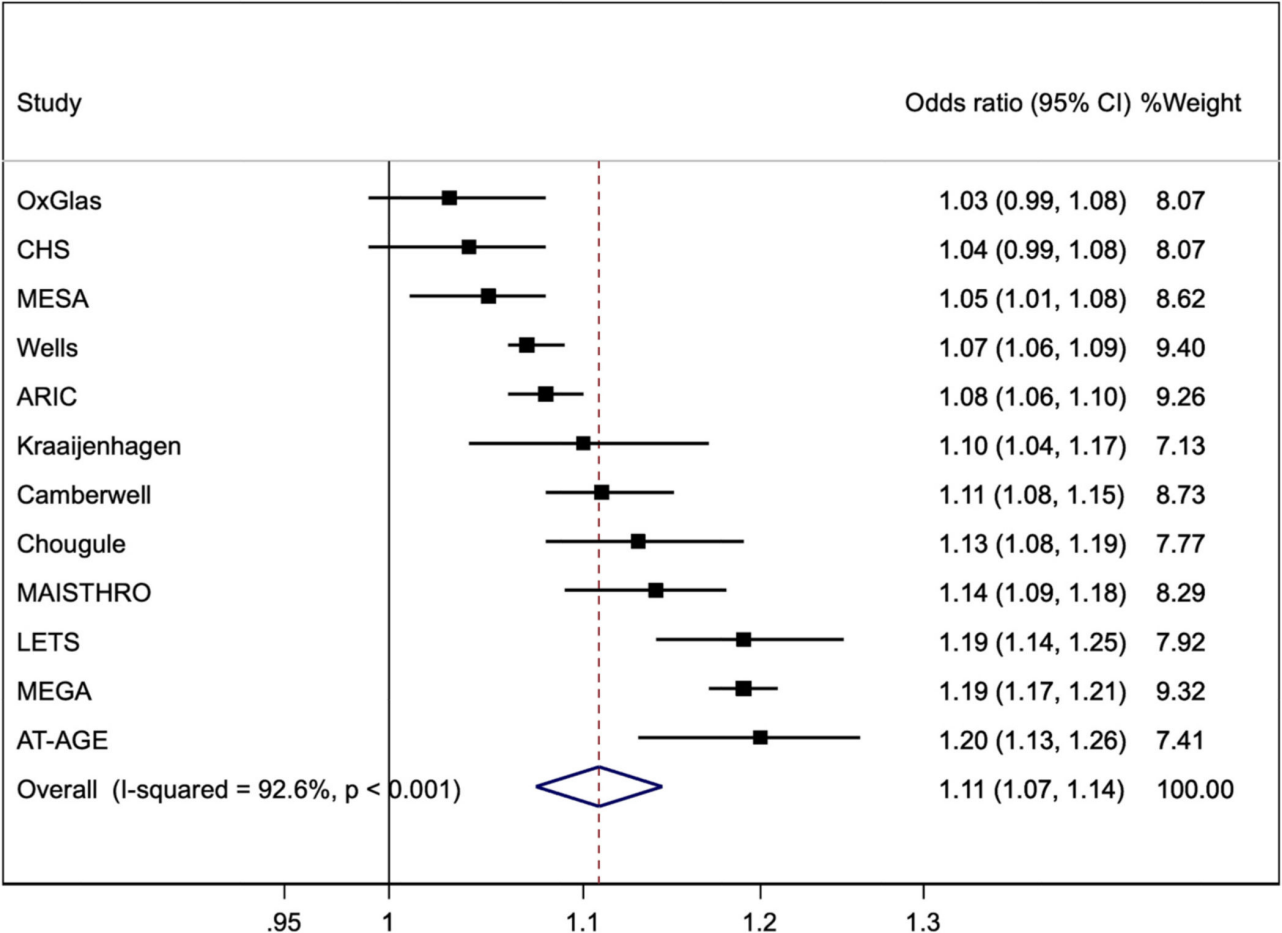
Odds ratios (95%CI) per 10 iu/dl higher Factor VIII (except that results for CHS, ARIC and MESA are hazard ratios). Results for Kraaijenhagen, MAISTHRO and Wells are estimated from other published data. Results are adjusted for age, sex and BMI except OxGlas (women only) was adjusted for age and BMI; Wells adjusted for age and sex; Kraaijenhagen adjusted for age, sex, fibrinogen, CRP and homocysteine; MAISTHRO adjusted for age, sex, BMI and oral contraceptive use; and MESA adjusted for age, sex, BMI, ethnicity and education.

Fig. 3 shows the analysis of FVIII per 10 IU/dl categories, performed in the seven studies noted above and in five other published case-control studies [12,23–26]. The pooled odds ratio of VTE was 1.11 (1.07, 1.14).

**Fig. 4.**
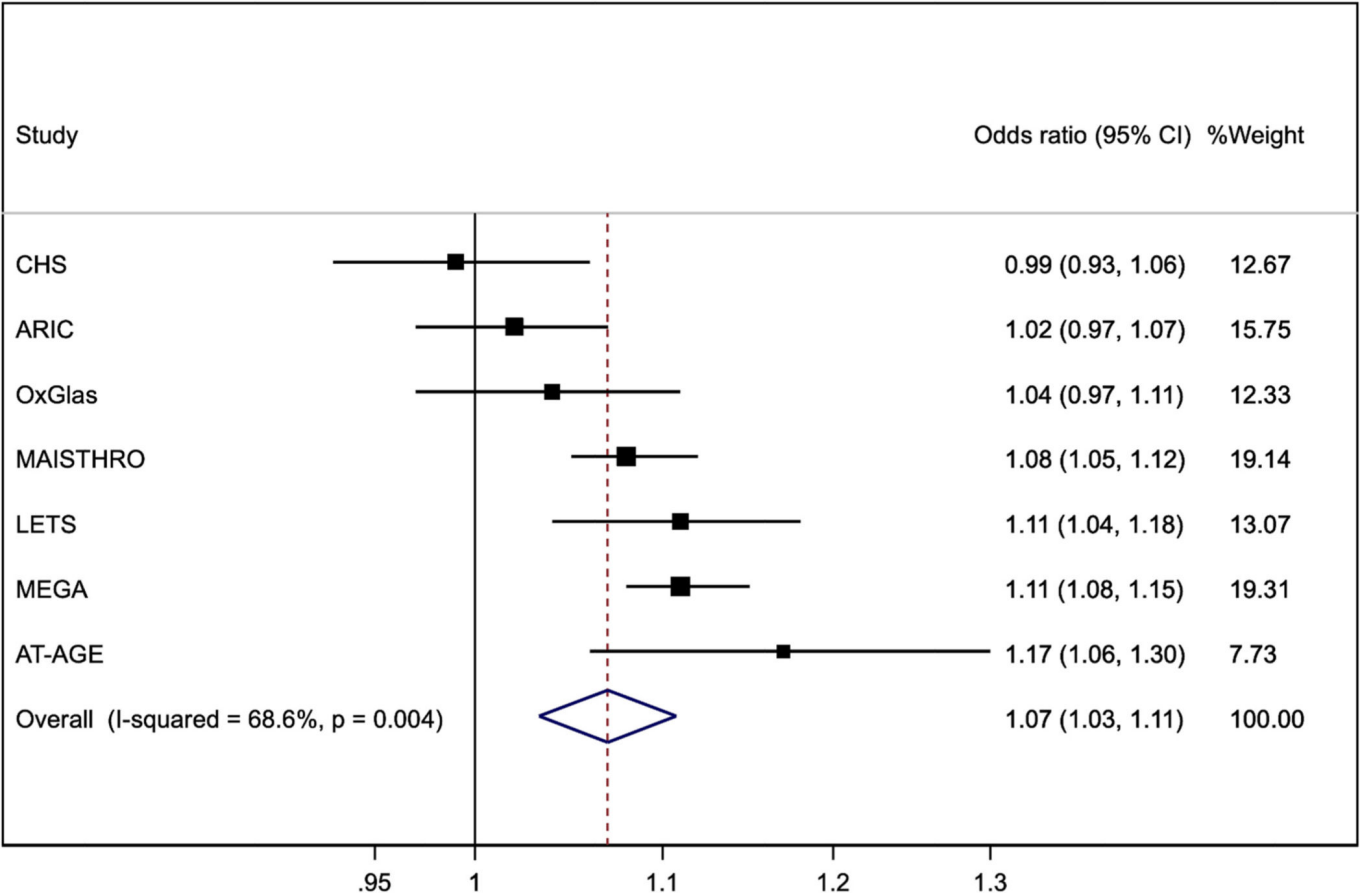
Odds ratios (95%CI) per 10 iu/dl higher Factor IX. Results for MAISTHRO are estimated from other published data. Results are adjusted for age, sex and BMI except that OxGlas (women only) adjusted for age and BMI and MAISTHRO adjusted additionally for oral contraceptive use.

Fig. 4 shows the corresponding analysis of FIX, performed in six of the seven studies noted above, and one other case-control study [24]. The pooled odds ratio of VTE was 1.07 (1.03, 1.11).

### 3.5. Analysis by greater than 90th percentile threshold versus less

**Fig. 5.**
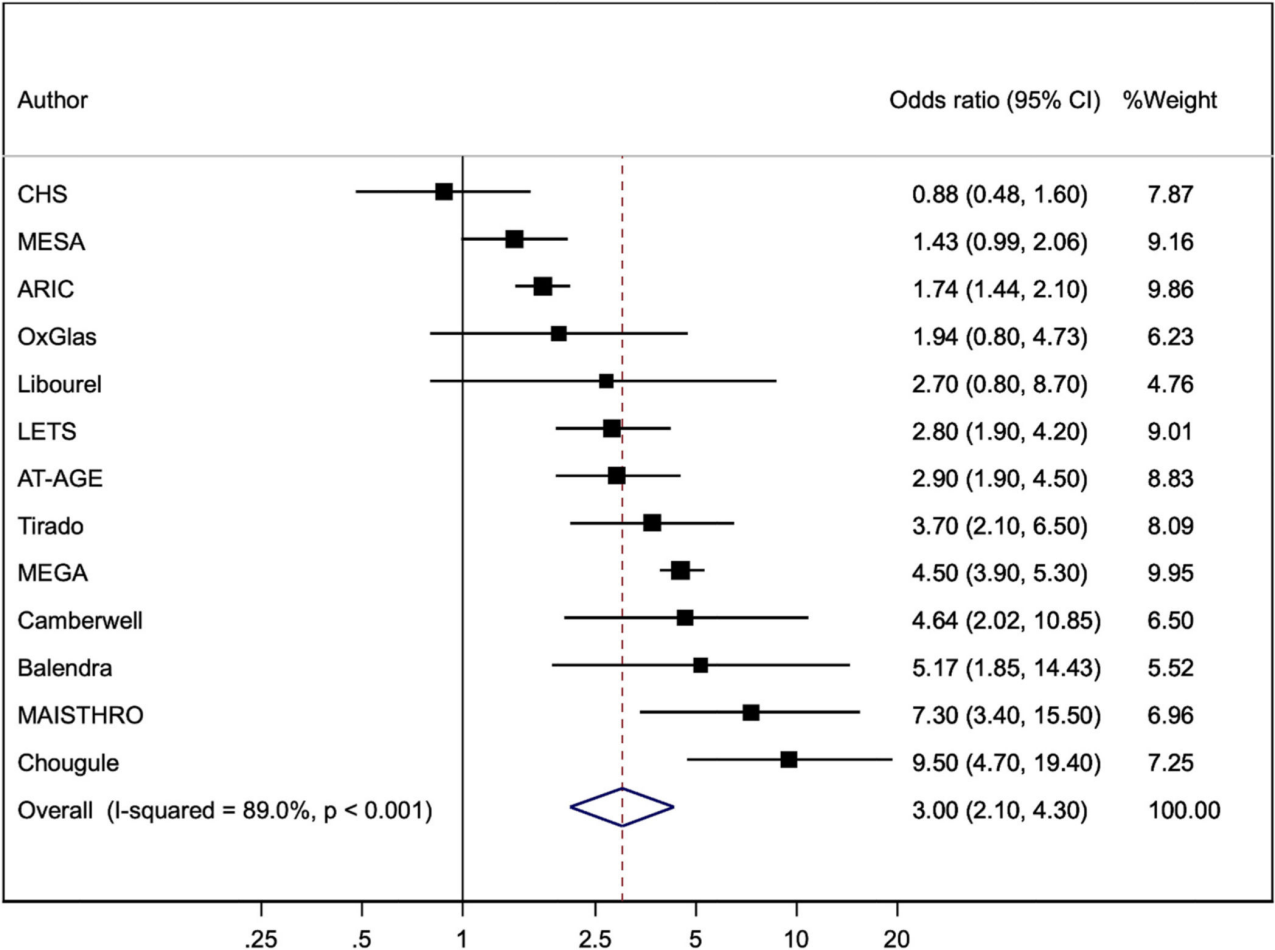
Odds ratios (95%CI) comparing greater than, to less than, the 90th percentile threshold of FVIII (except that results for CHS, ARIC AND MESA are hazard ratios). Results are adjusted for age, sex and BMI except that OxGlas (women only) adjusted for age and BMI; MAISTHRO adjusted additionally for oral contraceptive use; Camberwell and Chougule adjusted for age and sex; Libourel adjusted for age, sex, hormones and thrombophilias; Tirado adjusted for age, sex and thrombophilias; and MESA adjusted for age, sex, BMI, ethnicity and education.

Fig. 5 shows the analysis of FVIII by 90th percentile threshold, performed in six of the seven studies noted above, and in seven other case-control studies [9,24–28]. The pooled odds ratio of VTE was 3.00 (2.10, 4.30).

**Fig. 6.**
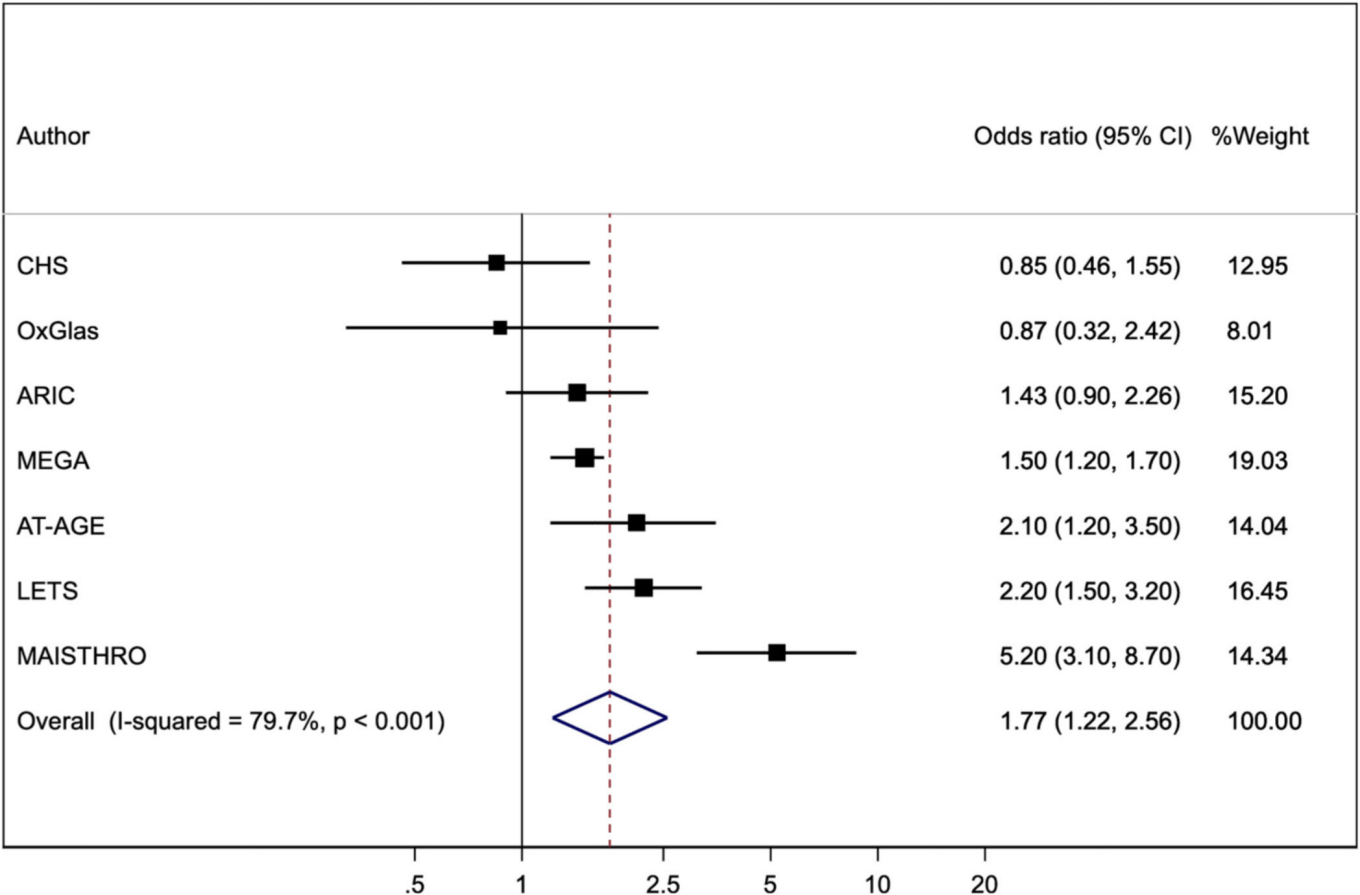
Odds ratios (95%CI) comparing greater than, to less than, the 90th percentile threshold of FIX. Results are adjusted for age, sex and BMI except that OxGlas (women only) adjusted for age and BMI and MAISTHRO adjusted additionally for oral contraceptive use.

Fig. 6 shows the analysis for FIX, performed in six of the seven studies noted above, and in one other case-control study [24]. The pooled odds ratio of VTE was 1.77 (1.22, 2.56).

**Fig. 7.**
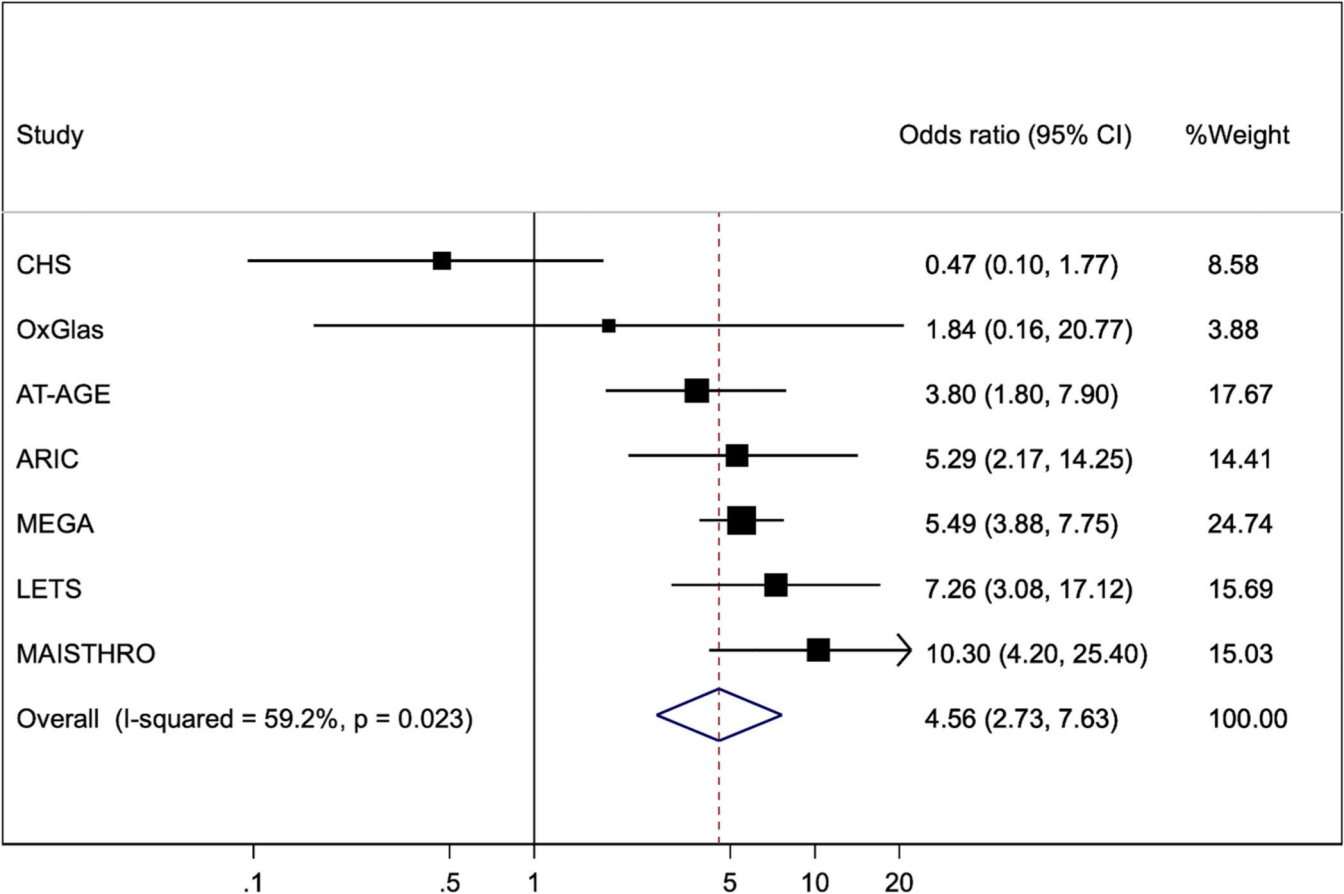
Odds ratios (95 % confidence intervals) for joint analysis of Factor VIII and Factor IX by 90th percentile thresholds. The odds ratios compare those with both factors above their 90 % threshold versus those with neither above that threshold. All studies adjusted for age and BMI; all but OxGlas (in women) adjusted for sex; MAISTHRO adjusted additionally for oral contraceptive use. Furthermore, MAISTHRO’s results are evaluated when Factors XI and XII are below their 90 % threshold.

Fig. 7 shows the joint analysis of FVIII and FIX, also performed in six of the seven studies noted above, and in one other case-control study [24]. The pooled odds ratio was 4.56 (2.73, 7.63).

### 3.6 Further analyses

A quality assessment of the papers selected for quantitative meta-analyses is presented in Supplemental Table 3 (Ottawa-Newcastle scores). Quality was generally high. A PRISMA checklist for reporting systematic review or meta-analysis appears in Supplemental Table 4.

In all four types of analysis, there were significant associations of FVIII and VTE risk both in the pooled three cohort studies, and in the pooled case-control studies. The pooled hazard ratios for the case-control studies were 2–3 times higher compared to the odds ratios for the cohort studies. Details are given in Supplemental Table 5.

## 4. Discussion and conclusions

In this systematic review and meta-analysis, we analysed both dose-response (per quarter, and per one SD increase); and threshold analyses of levels above the 90th percentile. We report that VTE risk increases with higher levels of both FVIII and FIX, with a stronger and more consistent association for FVIII. While there was good evidence of linearity for FVIII, the patterns of odds ratios by quarters of FIX suggest a non-linear threshold effect. These results are consistent with the findings of the largest study, MEGA [16], that FVIII shows a stronger association with VTE risk than FIX; and that for each factor the relative risk increases to a maximum at the highest percentile cut-off levels. There does not appear to be a threshold of factor levels at which risk estimates do not further increase.

We included studies from several European countries, North America, and India; and covered the whole adult age range of 17–100 years, in both men and women. Data were adjusted for age, sex and obesity; and ethnicity where applicable: which are possible confounders. Further cohort studies would be useful, to compare with case-control studies which are the majority of published studies to date, and which showed higher odds ratios for FVIII and VTE risk compared to the hazard ratios in cohort studies in the present analysis (Supplemental Table 5).

Possible reasons for the lower odds ratios in cohort studies, which measured FVIII years before most VTE episodes occurred, include that they assume that baseline levels of haemostatic factors remain constant over time. Variation in FVIII levels over time results in regression dilution in both cohort and case-control studies, related to within-person short-term variability plus laboratory analytic variability, but cohort studies have additional within-person variability due to change over time. This may lead to under-estimation of FVIII’s associations with VTE in cohort studies, due to regression dilution. We suggest that in future studies, serial measurement, and regression dilution estimates, would be useful.

In contrast, most case-control studies of VTE assayed FVIII level 3–6 months after completion of a course of anticoagulant therapy of 3–6 months duration, hence within a year of the event. While they assume that this time period might allow resolution of acute-phase reactant increases in FVIII; it is possible that FVIII levels might remain higher due to ongoing subclinical thrombosis (reverse causality): which is suggested by the associations of FVIII with increased risk of recurrent VTE (see below) This might result in over-estimation of its associations with first VTE in case-control studies.

At the date of our literature search, there were two nested case-control studies of FIX and VTE risk [18] but no cohort studies. The first population-based cohort study of FIX was recently published [29]: it reported significant associations for each of FVIII and FIX after risk factor adjustment; but after adjustment for FVIII the association of FIX with VTE was attenuated. While the larger MEGA study reported that the association of FIX with VTE remained significant after adjustment for FVIII [16], further cohort studies of FIX and FVIII would be useful to confirm this finding.

It is useful to define thresholds for high levels at which a clinically significant risk is identified: for example, doubling of risk compared to levels below the threshold. The LETS study used the 90th percentile threshold for FVIII and VTE risk [10]; then reported a joint effect on VTE risk of FVIII and FIX levels above their 90th percentiles [14]. We therefore performed additional analyses of these higher level effects on VTE risk in six of our previous studies [15,16,18,20,21]; and included such analyses where these were presented in six other studies of FVIII; and in one other study of FIX and its interaction with FVIII. The pooled odds ratio was 3.00 (2.10, 4.30) for FVIII (Fig. 5); 1.77 (1.22, 2.56) for FIX (Fig. 6); and 4.56 (2.73, 7.63) for their joint association (Fig. 7). These associations suggest that the 90 % thresholds of FVIII and FIX, and their joint effect, might be useful for identifying persons at clinically significant increased risk of VTE. However, further studies are required to assess their predictive value.

FVIII and VWF (usually assayed as VWF antigen, VWF:Ag) circulate as a high-affinity non-covalent complex [30]; hence their plasma levels are highly correlated in population samples, and VWF level may be the driver of the association of FVIII with risk of thrombosis [31]. Some studies reported that FVIII and VWF showed independent associations with VTE risk [18,32]; but others did not confirm this [10,16]. Further studies are required to establish their relative contributions.

It is important to consider whether the associations of FVIII and FIX levels with VTE risk might be attributable to inflammatory diseases. FVIII and its carrier protein VWF in particular are reactant proteins. Their plasma levels increase significantly during acute phase responses to trauma, surgery, acute inflections and physical or psychological stress, and may persist for several days or weeks [33–35]. Their levels also increase significantly and persistently in chronic conditions, including pregnancy, malignancies, chronic infections, and chronic inflammatory diseases – including rheumatoid arthritis, systemic lupus erythematosus and other connective tissue diseases, and chronic graft-versus-host disease [36]. VWF levels correlate with activity of some of these chronic diseases [36]. The effect of anti-inflammatory drugs on FVIII and VWF levels is not established.

A review of earlier studies of FVIII levels and VTE risk suggested that adjustment for the acute-phase reactant proteins C-reactive protein (CRP) or fibrinogen, did not account for the association of FVIII with VTE risk [33]. In our meta-analysis, five of the major studies [10,12,16,20,24], which comprised 71 % of the cases of FVIII and VTE risk and 87 % of the cases of FIX and VTE risk, reported no effect of CRP or fibrinogen levels on these associations. The largest study (MEGA) adjusted additionally for major illness (liver disease, kidney disease, rheumatoid arthritis, multiple sclerosis, heart failure, and cardiovascular diseases) and reported no effect of this further adjustment on the association of FVIII and VTE risk [16]. The British Regional Heart Study also reported no association of any inflammatory marker (C-reactive protein, fibrinogen, white cell count, plasma viscosity, interleukin-6) on the association of FVIII with risk of VTE [29]. While these studies suggest that the associations of FVIII and FIX with VTE risk we report in this meta-analysis are not significantly affected by acute phase reactants or major illnesses, we recommend that future studies should routinely adjust for these potential confounders.

The results of our meta-analysis suggest that persons who have levels of FVIII or FIX above the 90th percentile thresholds have an increased risk of VTE: approximately twofold for FIX, threefold for FVIII, and fivefold for high FVIII and FIX together. While potentially useful in adding to current VTE risk scores, an important practical issue is the need for specific age, sex, ethnicity, hormone use and obesity reference ranges for FVIII and FIX levels [5–8,23–25]. Ideally, these should be calculated from local population samples, as mean levels vary internationally [37].

Mass population screening by activity assays would be expensive; and (like other thrombophilias) potentially futile. The most promising indication for testing of FVIII and FIX levels may be added prediction of recurrent VTE. Our literature search produced 6 cohort studies of recurrent FVIII, [13,38–43] which merit additional studies and a future meta-analysis. FVIII has already been included in development of two risk scores for recurrent VTE [38,43].

The genetic basis of increased FVIII and FIX levels is largely unknown, apart from the ABO blood group [17]. There are only a few cases of well-defined gene lesions that were associated with increased levels [44–46]. Further studies are required to establish the degree to which the associations of levels are due to genetic factors.

Strengths of our study include: identification of an adequate number of studies which met our predefined criteria (5327 VTE cases with FVIII levels, and 3498 cases with FIX levels); additional analyses of seven of our own studies to facilitate our statistical analyses; and inclusion of diverse studies performed in Europe, North America and India with representation of the whole adult age range, men and women, and several ethnic groups.

Although this study provides the most comprehensive quantitative summary of the associations between FVIII and FIX with VTE, our meta-analyses have limitations. Statistical limitations include the relatively small number of studies identified and the heterogeneity in effect sizes, due in part to mixing individual participant and published data, variations in study design, the lack of a common metric used to measure relative risk and the varying adjustments made in the fitted models per study. Clinical limitations include the need to confirm or refute the potential predictive value of FVIII and FIX levels in further cohort studies, the heterogeneity of FVIII and FIX assays, and the need for further genetic studies (in addition to ABO blood group) to assess potential causality.

We conclude that risk of VTE increases across population distributions of FVIII and FIX levels. Levels above the 90th percentile have almost twice the risk for FIX level compared to levels below; three-fold risk for FVIII level; and almost five-fold risk for both FVIII and FIX levels elevated. Further population studies are suggested to assess potential predictive value and causality.

## Supplementary Material

1

## Figures and Tables

**Table 1:** Characteristics of Published Studies

A. Characteristics of 15 published studies of FVIII and VTE risk^a^						

Study (reference)	Location	Age, sex	Study type	Cases/controls^b^	Assay	Multivariable adjustments

Balendra 1991 [9]	UK	17–77, M + F	Case-control, VTE	84/47	Activity	Age, sex, BMI
^a^LETS 1995 [10]	Netherlands	17–73, M + F	Case-control, first VT	301/301	Activity	Age, sex, BMI, CRP
Kraaijenhagen 2000 [12]	Netherlands	67 (17), M + F	Case-control, VTE	165/60	Activity	Age, sex, CRP, fgen
^a^OxGlas 2000 [15]	UK	45–64, F	Case-control, VTE	66/163	Activity	Age, BMI
Libourel 2002 [29]	Netherlands	15–81, M + F	Case-control, VTE	17/36	Activity	Age, sex, hormones, thrombophilia
^a^ARIC 2002 [18]	USA	45+, M + F	Cohort, first VTE	895/15,174	Activity	Age, sex, BMI, ethnicity, CRP, fgen
^a^CHS 2002 [18]	USA	65–100, M + F	Cohort, first VTE	136/4749	Activity	Age, sex, BMI, ethnicity, CRP, fgen
Camberwell 2003 [25]	UK	19–81, M + F	Case-control, VTE	100/100	Activity	Age, sex
Tirado 2005 [27]	Spain	42 (14), M + F	Case-control, VTE	250/250	Activity	Age, sex, thrombophilias
Wells 2005 [23]	Canada	56 (15), M + F	Case-control, VTE	300/300	Activity	Age, sex
MAISTHRO 2009 [24]	Germany	18–75, M + F	Case-control, VTE	198/499	Activity	Age, sex, BMI, OC, fgen
Chougule 2016 [26]	India	18–59, M + F	Case-control, VTE	101/86	Activity	Age, sex
^a^MEGA 2019 [16]	Netherlands	18–70, M + F	Case-control, first VTE	2088/2910	Antigen	Age, sex, BMI, CRP, major illness
^a^AT-AGE 2021 [21]	Netherlands, USA	70–100, M + F	Case-control, first VTE	401/431	Activity	Age, sex, BMI, centre
^a^MESA 2021 [20]	USA	45–84, M ± F	Cohort, VTE	225/6689	Activity	Age, sex, BMI, ethnicity, education, centre
Total				5327/31,795		

B. Characteristics of 7 published studies of FIX and VTE risk^a^						

Study (reference)	Location	Age, sex	Study type	Cases/controls	Assay	Multivariable adjustments

^a^LETS 2000 [14]	Netherlands	17–73, M + F	Case-control, first VT	301/301	Activity	Age, sex, BMI, CRP
^a^OxGlas 2000 [15]	UK	45–64, F	Case-control, VTE	65/162	Activity	Age, BMI
^a^ARIC 2009 [19]	USA	45–64, M + F	Nested case-control, VTE	274/653	Antigen	Age, sex, BMI, ethnicity, CRP, fgen
^a^CHS 2009 [19]	USA	65–100, M + F	Nested case-control, VTE	186/386	Antigen	Age, sex, BMI, ethnicity, CRP, fgen
MAISTHRO 2009 [24]	Germany	18–75, M + F	Case-control, VTE	183/499	Activity	Age, sex, BMI, OC, fgen
^a^MEGA 2019 [16]	Netherlands	18–70, M + F	Case-control, first VTE	2088/2910	Antigen	Age, sex, BMI, CRP, major illness
^a^AT-AGE 2021 [21]	Netherlands, USA	70–100, M ± F	Case-control, first VTE	401/431	Activity	Age, sex, BMI, centre
Total				3498/5342		

Abbreviations. ABO: blood group, ARIC: Atherosclerosis Risk In Communities study, BMI: body mass index, CHS: Cardiovascular Health Study, CRP: C-reactive protein, fgen: fibrinogen, LETS: Leiden Thrombophilia Study, MAISTHRO: Main Isar Thrombose registry study, MEGA: Multiple Environmental and Genetic Assessment of risk factors for VT study, MESA: Multi-Ethnic Study of Atherosclerosis, OC: oral contraceptives, OxGlas: Oxford Glasgow study.

a Studies where data re-analysed.

b For the three cohort studies, cases/non-cases.
