# Supplementary material for: Plasma levels of coagulation factors VIII and IX and risk of venous thromboembolism: Systematic review and meta-analysis
Source: Thromb Res. Author manuscript; Available in PMC 2024 Feb 21. (PMC10881212; doi:10.1016/j.thromres.2023.06.026)

**FACTORS VIII AND IX AND VTE RISK –**

**SUPPLEMENTAL 1 MAR 2023**

**Supplemental Table 1. Search terms.**

Factor VIII or FVIII

and

Thromb* or Lung embolism or pulmonary embolism

and

Cohort studies [MeSH] or prospective studies [MeSH] or retrospective studies [MeSH] or case-control study [MeSH] or epidemiologic studies [MeSH] or cohort stud* or prospective stud* or population stud* or case-control stud* or observational stud* or retrospective stud* or longitudinal stud* or odds or risk or hazard

Factor IX or FIX

and

Thromb* or Lung embolism or pulmonary embolism

and

Cohort studies [MeSH] or prospective studies [MeSH] or retrospective studies [MeSH] or case-control study [MeSH] or epidemiologic studies [MeSH] or cohort stud* or prospective stud* or population stud* or case-control stud* or observational stud* or retrospective stud* or longitudinal stud* or odds or risk or hazard

**Supplemental Table 2. Selected percentiles in 6 selected studies for FVIII and FIX levels (IU/dl)**

Percentile ARIC CHS LETS MEGA AT-AGE OxGlas

FVIII

25 109 97 99 86 112 109

50 129 119 119 109 132 139

75 157 146 143 139 152 184

90 184 175 166 173 176 226

FIX

25 119 129 89 92 104 113

50 138 148 101 103 117 138

75 160 168 114 116 132 164

90 182 192 129 129 143 189

**Supplemental Table 3. Quality assessment of papers selected for quantitative meta-analyses (Ottawa-Newcastle scores).**

**FVIII Selection Comparability Outcome/Exposure**

Max **** Max ** Max ***

Cohort Studies

ARIC 2002^18^  **** ** ***

CHS 2002^18^ **** ** ***

MESA 2021^20^ **** ** ***

Case-control studies

Balendra, 1991^9^  *** ** ***

LETS, 1995^10^ **** ** ***

Kraajenhaagen 2000^12^ *** ** ***

OxGlas 2000^15^ *** ** ***

Libourel 2002^28^  ** ** ***

Camberwell 2003^25^  *** ** ***

Tirado 2005^27^ **** ** ***

Wells 2005^23^ **** ** ***

MAISTHRO 2009^24^ *** ** ***

Chougule 2016^26^ *** ** ***

MEGA 2019^16^ **** ** ***

AT-AGE 2021^21^ **** ** ***

Note: For Balendra,^9^ MD thesis, University of Belfast, 1990 also reviewed.

**FIX Selection Comparability Outcome/Exposure**

Nested Case- Control Studies

ARIC 2002^18^  **** ** ***

CHS 2002^18^ **** ** ***

Case-control studies

LETS, 1995^10^ **** ** ***

OxGlas 2000^15^ *** ** ***

MAISTHRO 2009^24^ *** ** ***

MEGA 2019^16^ **** ** ***

AT-AGE 2021^21^ **** ** ***

**Supplemental Table 4. PRISMA checklist.**

Text S1 - Checklist of items to include when reporting a systematic review or meta-analysis

| Section/topic | # | Checklist item | Reported on page # |
| --- | --- | --- | --- |
| **TITLE** | | | |
| Title | 1 | Identify the report as a systematic review, meta-analysis, or both. | # 1 |
| **ABSTRACT** | | | |
| Structured summary | 2 | Provide a structured summary including, as applicable: background; objectives; data sources; study eligibility criteria, participants, and interventions; study appraisal and synthesis methods; results; limitations; conclusions and implications of key findings; systematic review registration number. | # 2-4 |
| **INTRODUCTION** | | | |
| Rationale | 3 | Describe the rationale for the review in the context of what is already known. | # 2-3 |
| Objectives | 4 | Provide an explicit statement of questions being addressed with reference to participants, interventions, comparisons, outcomes, and study design (PICOS). | # 3-4 |
| **METHODS** | | | |
| Protocol and registration | 5 | Indicate if a review protocol exists, if and where it can be accessed (e.g., Web address), and, if available, provide registration information including registration number. | # 3 |
| Eligibility criteria | 6 | Specify study characteristics (e.g., PICOS, length of follow-up) and report characteristics (e.g., years considered, language, publication status) used as criteria for eligibility, giving rationale. | # 3-4 |
| Information sources | 7 | Describe all information sources (e.g., databases with dates of coverage, contact with study authors to identify additional studies) in the search and date last searched. | # 3-4 |
| Search | 8 | Present full electronic search strategy for at least one database, including any limits used, such that it could be repeated. | # Suppl. Table 1 |
| Study selection | 9 | State the process for selecting studies (i.e., screening, eligibility, included in systematic review, and, if applicable, included in the meta-analysis). | Suppl. Figures 1 and 2 |
| Data collection process | 10 | Describe method of data extraction from reports (e.g., piloted forms, independently, in duplicate) and any processes for obtaining and confirming data from investigators. | # 3-4 |
| Data items | 11 | List and define all variables for which data were sought (e.g., PICOS, funding sources) and any assumptions and simplifications made. | # 3-4 |
| Risk of bias in individual studies | 12 | Describe methods used for assessing risk of bias of individual studies (including specification of whether this was done at the study or outcome level), and how this information is to be used in any data synthesis. | # 3, Ottawa-Newcastle scores |
| Summary measures | 13 | State the principal summary measures (e.g., risk ratio, difference in means). | # 4, Hazard/odds ratios |
| Synthesis of results | 14 | Describe the methods of handling data and combining results of studies, if done, including measures of consistency (e.g., I^2^) for each meta-analysis. | # 3-4; I^2^ |
| Risk of bias across studies | 15 | Specify any assessment of risk of bias that may affect the cumulative evidence (e.g., publication bias, selective reporting within studies). | # 3, Ottawa-Newcastle scores |
| Additional analyses | 16 | Describe methods of additional analyses (e.g., sensitivity or subgroup analyses, meta-regression), if done, indicating which were pre-specified. | # 6, comparison of cohort and case-control studies |
| RESULTS | | | |
| Study selection | 17 | Give numbers of studies screened, assessed for eligibility, and included in the review, with reasons for exclusions at each stage, ideally with a flow diagram. | Suppl. Figures 1 and 2 |
| Study characteristics | 18 | For each study, present characteristics for which data were extracted (e.g., study size, PICOS, follow-up period) and provide the citations. | Table 1 |
| Risk of bias within studies | 19 | Present data on risk of bias of each study and, if available, any outcome-level assessment (see Item 12). | Suppl. Table 3 |
| Results of individual studies | 20 | For all outcomes considered (benefits or harms), present, for each study: (a) simple summary data for each intervention group and (b) effect estimates and confidence intervals, ideally with a forest plot. | Table 1, Figures 1-7, Suppl. Figures 3-7 |
| Synthesis of results | 21 | Present results of each meta-analysis done, including confidence intervals and measures of consistency. | Figures 1-7, Suppl. Figures 3-7 |
| Risk of bias across studies | 22 | Present results of any assessment of risk of bias across studies (see Item 15). | Suppl. Table 3 |
| Additional analysis | 23 | Give results of additional analyses, if done (e.g., sensitivity or subgroup analyses, meta-regression [see Item 16]). | Suppl. Figure 7 |
| DISCUSSION | | | |
| Summary of evidence | 24 | Summarize the main findings including the strength of evidence for each main outcome; consider their relevance to key groups (e.g., health care providers, users, and policy makers). | # 6-9 |
| Limitations | 25 | Discuss limitations at study and outcome level (e.g., risk of bias), and at review level (e.g., incomplete retrieval of identified research, reporting bias). | # 6-9 |
| Conclusions | 26 | Provide a general interpretation of the results in the context of other evidence, and implications for future research. | # 6-9 |
| FUNDING | | | |
| Funding | 27 | Describe sources of funding for the systematic review and other support (e.g., supply of data); role of funders for the systematic review. | # 9 |

**Supplemental Table 5.** **Comparison of pooled results from cohort and case-control studies of Factor VIII.**

|  | Cohort studies | |  | Case-control studies | |
| --- | --- | --- | --- | --- | --- |
| Analysis | n | HR (95%CI) |  | n | OR (95%CI) |
| Per 10 IU/dl | 3 | 1.25 (1.11, 1.39) |  | 7 | 1.58 (1.30, 1.92) |
| Per SD | 3 | 1.06 (1.04, 1.09) |  | 9 | 1.13 (1.08, 1.17) |
| Q4 *vs* Q1 | 3 | 1.77 (1.29, 2.42) |  | 4 | 4.63 (2.68, 8.01) |
| >90^th^ % *vs* below | 3 | 1.43 (1.04, 1.98) |  | 10 | 4.00 (3.10, 5.15) |

HR = hazard ratio; OR = odds ratio; CI = confidence interval


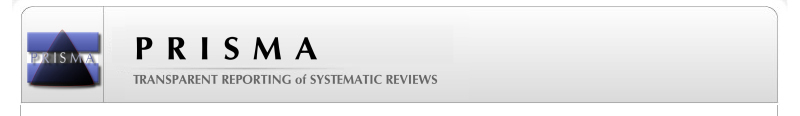
**Supplemental Figure 1. PRISMA 2009 Flow Diagram FVIII-VTE**

Full-text articles excluded, with reasons
(n = 50)

-pr

Studies included in qualitative synthesis
(n = 21)

Additional records identified through other sources
(n = 2)

Records identified through database searching
(n = 786)

Studies included in quantitative synthesis (meta-analysis)
(n =15)

Full-text articles assessed for eligibility
(n = 71)

Records excluded
(n = 715)

Records screened
(n = 786)

Records after duplicates removed
(n = 786)

## Identification

## Eligibility

## Included

## Screening

**Supplemental Figure 2. PRISMA 2009 Flow Diagram FIX-VTE**

Studies included in quantitative synthesis (meta-analysis)
(n = 7 )

Records excluded
(n = 176 )

Records screened
(n = 205 )

Records after duplicates removed
(n = 205 )

## Identification

## Eligibility

## Included

## Screening

Additional records identified through other sources
(n = 0 )

Records identified through database searching
(n = 205)

Full-text articles excluded, with reasons
(n =18 )

Full-text articles assessed for eligibility
(n = 29 )

Studies included in qualitative synthesis
(n = 11 )

*From:*  Moher D, Liberati A, Tetzlaff J, Altman DG, The PRISMA Group (2009). *P*referred *R*eporting *I*tems for *S*ystematic Reviews and *M*eta-*A*nalyses: The PRISMA Statement. PLoS Med 6(7): e1000097. doi:10.1371/journal.pmed1000097

**For more information, visit www.prisma-statement.org.**

**Supplemental Figure 3. Odds ratios (95% confidence intervals) by quarters (reference: first quarter) of FVIII and FIX, within studies (except that FVIII results for CHS, ARIC and MESA are hazard ratios)**.

Studies included are ARIC, CHS, LETS, MEGA, AT-AGE, OxGlas and (for FVIII only) MESA. All results are adjusted for age, sex and BMI, except that OxGlas (women only) adjusted for age and BMI; and ARIC, CHS and MESA adjusted additionally for ethnicity.

 
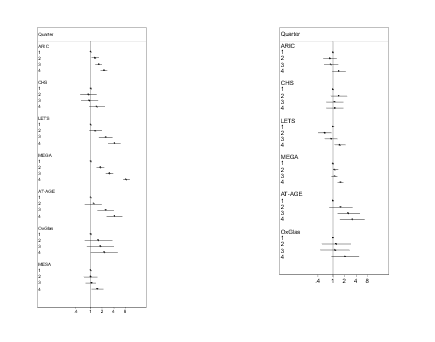


**Supplemental Figure 4. Pooled odds ratios (95% confidence intervals) by quarters of FVIII and FIX (base first quarter); note that the FVIII results for CHS, ARIC and MESA are hazard ratios.**

Studies included are ARIC, CHS, LETS. MEGA, AT-AGE, OxGlas and (for FVIII only) MESA. All results are adjusted for age, sex and BMI, except that OxGlas (women only) adjusted for age and BMI; and ARIC, CHS and MESA adjusted additionally for ethnicity.


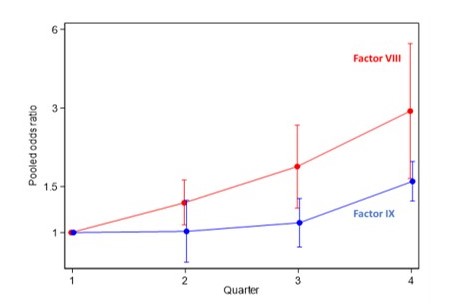


**Supplemental Figure 5. Odds ratios (95%CI) per standard deviation (SD) higher Factor VIII (except that results for CHS, ARIC and MESA are hazard ratios).**

Results for Kraaijenhagen, MAISTHRO and Wells are estimated from other published data.

**
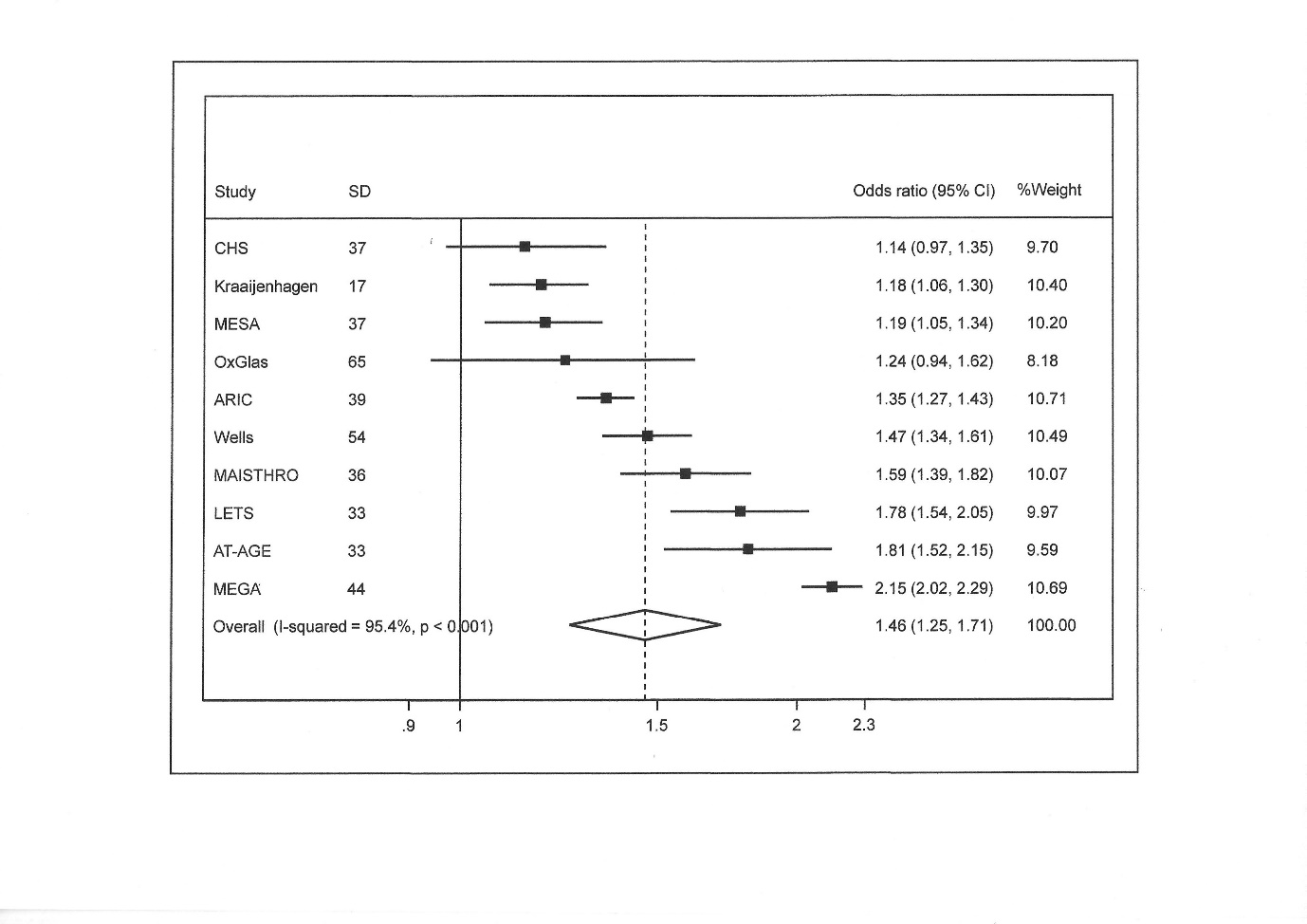
**

**Supplemental Figure 6. Odds ratios (95%CI) per standard deviation (SD) higher Factor IX.**

Results for MAISTHRO are estimated from other published data. Results are adjusted for age, sex and BMI except that OxGlas (women only) adjusted for age and BMI; ARIC and CHS adjusted additionally for ethnicity; and MAISTHRO adjusted additionally for oral contraceptive use.


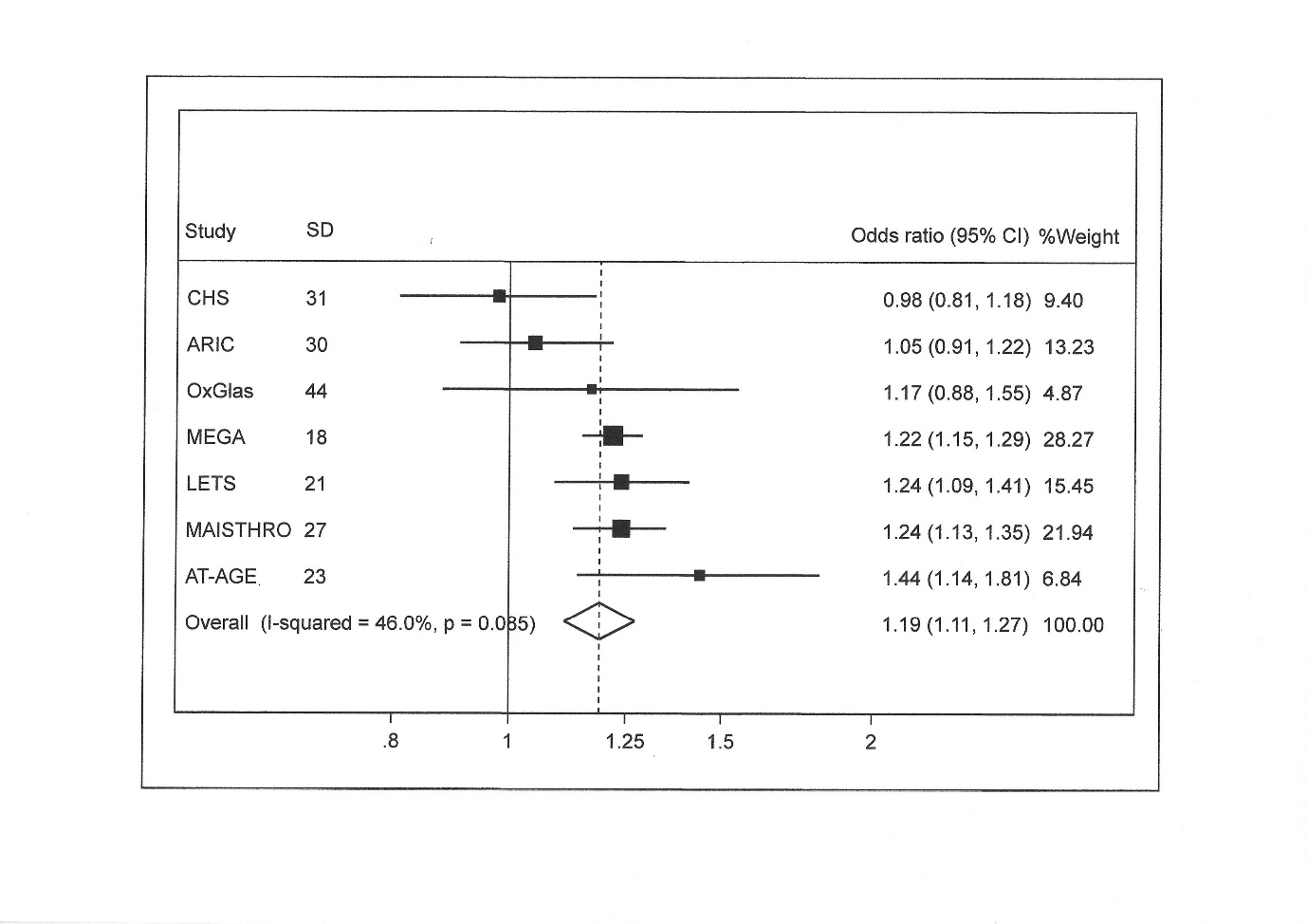

Supplement: 1 [file NIHMS1957297-supplement-1.docx]
